# Impact of Altitude Training on Athletes’ Aerobic Capacity: A Systematic Review and Meta-Analysis

**DOI:** 10.3390/life15020305

**Published:** 2025-02-17

**Authors:** Lin Deng, Yuhang Liu, Baili Chen, Jiawan Hou, Ao Liu, Xiaoyi Yuan

**Affiliations:** 1 College of Education, Beijing Sports University, Beijing 100084, China; denglin0912@bsu.edu.cn (L.D.); bsulyh@bsu.edu.cn (Y.L.); chen010504@bsu.edu.cn (B.C.); 2023210495@bsu.edu.cn (J.H.); 2023210499@bsu.edu.cn (A.L.); 2 State General Administration of Sport Key Laboratory of Sports Training, Beijing 100084, China

**Keywords:** altitude training, aerobic capacity, maximal oxygen uptake, hemoglobin, meta-analysis

## Abstract

**Purpose:** This study systematically evaluated the effects of altitude training on athletes’ aerobic capacity, focusing on optimal training modalities and intervention durations. **Methods:** Eight databases (CNKI, CSPD, PubMed, Ovid Medline, ProQuest, Cochrane Library, Embase, and Scopus) were searched for randomized controlled trials on altitude training and aerobic capacity following PRISMA guidelines, covering publications up to 15 October 2024. The risk of bias was assessed using Cochrane tools, and a meta-analysis was conducted using Review Manager 5.4 with a random-effects model. Sensitivity and subgroup analyses were performed to identify heterogeneity and influencing factors. **Results:** Thirteen studies involving 276 participants (aged 18–35) were included. Meta-analysis revealed that compared to low-altitude training, altitude training significantly increased hemoglobin (SMD = 0.7, 95% CI: 0.27–1.13, *p* = 0.03) and hemoglobin mass (SMD = 0.49, 95% CI: 0.1–0.89, *p* = 0.16) but had no significant effect on maximal oxygen uptake (SMD = −0.13, 95% CI: −1.21–0.96, *p* = 0.68). Altitude training also improved performance in trial tests (SMD = −28.73, 95% CI: −58.69–1.23, *p* = 0.002). Sensitivity analysis confirmed the robustness of hemoglobin and trial test results. Subgroup analysis showed that the “live high, train high” (LHTH) approach and interventions lasting longer than three weeks were most effective in enhancing aerobic capacity. **Conclusions:** Altitude training improves athletes’ aerobic capacity by enhancing hematological indicators and trial test performance, though its impact on maximal oxygen uptake is minimal. LHTH and interventions exceeding three weeks yield superior outcomes. However, the findings are limited by the number and quality of the available studies.

## 1. Introduction

Altitude training, also referred to as hypoxic training, involves athletes training either at high altitudes or within artificially induced hypoxic environments [1]. This concept was initially introduced by Soviet researchers in the 1950s, who observed that the human body undergoes physiological adaptations to the low-oxygen conditions prevalent in high-altitude settings. Training under such conditions stimulates both the respiratory and cardiovascular systems, thereby enhancing endurance performance. The remarkable success of East African athletes during the 1968 Olympic Games in Mexico City (altitude: 2250 m) drew significant global attention to altitude training as an effective method for improving aerobic endurance [2,3,4]. Since then, extensive research has been conducted to investigate its physiological effects. However, insufficient acclimatization or improper application of altitude training may result in adverse outcomes, including compromised blood circulation, muscle atrophy, increased susceptibility to infections, gastrointestinal disturbances, and hematuria [5].

Aerobic capacity refers to the ability to sustain muscular activity under aerobic conditions, particularly the muscles’ capacity to uptake and utilize oxygen efficiently. It is a fundamental physiological attribute essential for all types of sports, particularly endurance-based disciplines [6,7]. Previous research has demonstrated a positive correlation between aerobic capacity and the oxygen-carrying capacity of hemoglobin in human red blood cells, which facilitates oxygen transport to the heart and skeletal muscles [8]. The unique geographic and climatic conditions of high altitudes induce a series of adaptive changes in athletes’ physiological functions. As altitude increases, the oxygen content in the air decreases, creating a hypoxic environment [9,10]. In response, the human body stimulates the expression of erythropoietin (EPO) through hypoxia-inducible factors (HIF) in the kidneys. Once released into the bloodstream, EPO promotes the differentiation of erythrocyte precursor cells in the bone marrow into mature red blood cells [11]. The resulting increase in the red blood cell count enhances the blood’s oxygen-carrying capacity, thereby improving athletes’ aerobic performance. Furthermore, studies have shown that altitude training significantly elevates hemoglobin concentration and the total hemoglobin mass, providing an additional boost to aerobic capacity [12,13,14]. Research on the application of altitude training to enhance endurance athletes’ performance has been ongoing for nearly half a century [15,16,17,18]. In addition to traditional altitude training, advancements in physiological research and the development of simulated hypoxic chambers have facilitated the emergence of alternative approaches. These include Live-High-Train-Low (Hi-Lo) [19], Living High-Training High-Training-Low (Hi-Hi-Lo) [20], Live-Low-Train-High (Lo-Hi) [21], Intermittent Hypoxic Training (IHT) [22,23,24], Simulated Altitude Training [25], and Diversified Altitude Training [26]. Initially, altitude training was recognized as a beneficial strategy for enhancing athletic performance, leading to the development of various training protocols. The Live-High-Train-Low (Hi-Lo) protocol promotes hematological adaptations and enhances oxygen transport through altitude exposure while allowing athletes to maintain high-intensity training at low altitudes [27]. However, its effectiveness remains a subject of debate. The Live-Low-Train-High (Lo-Hi) protocol emphasizes the combination of low-altitude recovery with high-altitude training [7], yet its efficacy is influenced by training duration and intensity. The Live-High-Train-High-Train-Low (Hi-Hi-Lo) protocol integrates high-altitude living, high-altitude training, and low-altitude high-intensity training to optimize hypoxic adaptation [7]; however, limited research has been conducted on this approach, and its underlying mechanisms remain unclear. Intermittent Hypoxic Training (IHT) involves a periodic exposure to hypoxic conditions to enhance aerobic capacity and mitigate the physiological stress associated with altitude acclimatization [28], but individual variability in response to this method is considerable. Despite these advancements, there is still no consensus regarding the optimal duration, intensity, and mode of altitude training interventions.

This study systematically reviewed and conducted a meta-analysis to evaluate the effects of altitude training on blood biomarkers associated with aerobic endurance and maximal oxygen uptake in athletes. Particular attention was given to the physiological mechanisms underlying blood and muscle adaptations induced by altitude training and their impact on athletic performance. The study aimed to provide novel insights into the optimal modalities and duration of altitude training while addressing common misconceptions in training practices. Additionally, this study sought to identify the most effective intervention strategies and offer evidence-based recommendations for tailoring altitude training programs to meet the individualized needs and performance objectives of athletes.

## 2. Materials and Methods

This study adhered to the guidelines of the Preferred Reporting Items for Systematic Reviews and Meta-Analyses (PRISMA). The risk of bias was evaluated using Review Manager 5.3 software. A fixed-effects model was applied, and the results were calculated using means and pre-intervention standard deviations (SD). The standardized mean difference (SMD) was reported with 95% confidence intervals (CIs). The study protocol has been registered with PROSPERO (CRD42023401488).

### 2.1. Search Strategy

A comprehensive literature search was conducted in the CNKI, CSPD, PubMed, Ovid Medline, ProQuest, Cochrane Library, Embase, and Scopus databases. The search targeted randomized controlled trials (RCTs) investigating the effects of altitude training interventions on aerobic capacity, covering studies published from the inception of each database until 15 October 2024. The search was conducted using the following terms: “Altitude Training”, “Plateau Training”, “Highland Training”, “Hypoxia Training”, “Maximum Oxygen Uptake”, “VO_2_max”, “Aerobic Capacity”, “Hemoglobin”, “Erythropoietin”, “EPO”, and “Athlete”. Boolean logic was applied to combine these keywords. A retrospective approach was also employed to ensure the comprehensive inclusion of relevant studies. The preliminary search yielded a total of 11,305 articles in both Chinese and English. Additionally, a manual search was performed to review the reference lists of included studies to identify further relevant articles. The electronic search was systematically conducted across the specified databases by two independent researchers. In cases of disagreement, a third reviewer was consulted to reach a final decision.

### 2.2. Inclusion/Exclusion Criteria

#### 2.2.1. Inclusion Criteria

The inclusion criteria for the literature were based on the PICOS principle in Cochrane systematic reviews:

(1) Study subjects: Athletes engaged in any sport, aged 18 years or older.
(2) Intervention: Any form of altitude endurance training.
(3) Experimental and control groups: The experimental group received any form of altitude training, while the control group underwent endurance training at sea level.
(4) Outcome indicators: Studies must report at least one of the following: maximal oxygen uptake, field test performance, blood markers, etc.
(5) Experimental design: Randomized controlled trials (RCTs) with a detailed exercise intervention program.

#### 2.2.2. Exclusion Criteria

The exclusion criteria for the study were as follows:

(1) Non-athlete subjects, including individuals with cardiorespiratory diseases.
(2) Studies without an aerobic training group (positive control group).
(3) The absence of pre- and post-intervention study parameters.
(4) Animal-based experiments.
(5) Literature not based on direct experimentation or relying on secondary citations of experimental data for review or analysis purposes.
(6) Studies lacking a clear description of experimental protocols, procedures, data collection, statistical testing, or analytical methods.
(7) Conference abstracts, review articles, dissertations, or qualitative studies.

**Figure 1:**
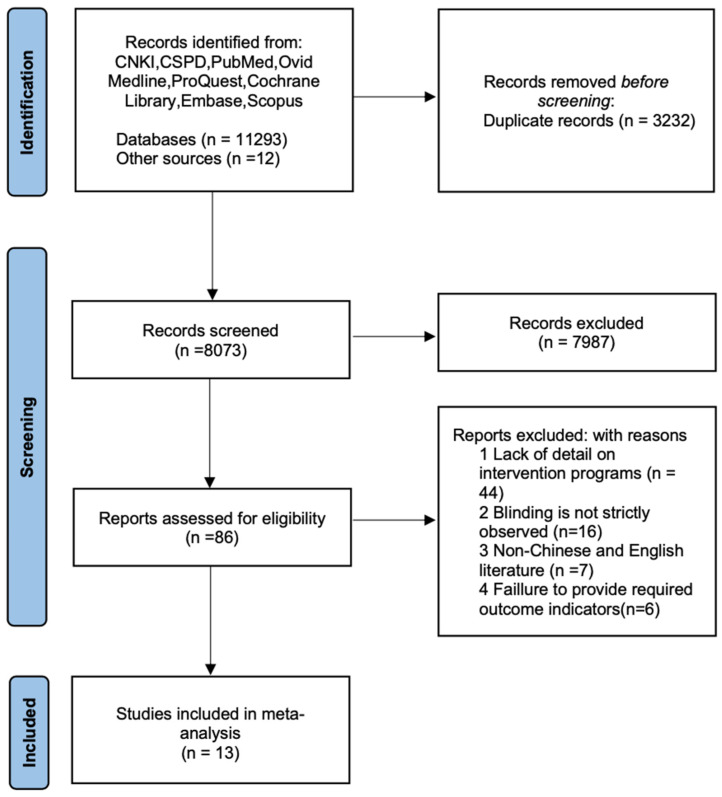
Literature screening.

Two researchers independently reviewed and evaluated each article according to the inclusion and exclusion criteria. Disagreements were resolved through discussion with a third researcher to ensure consistency and objectivity in the final selection. The study selection process is depicted in Figure 1, following PRISMA guidelines.

### 2.3. Screening of Literature

Literature de-duplication was performed using NoteExpress 3.6.0 software. Two researchers independently conducted the initial screening and full-text selection based on the predefined inclusion and exclusion criteria. The study selection process is presented in Figure 1.

### 2.4. Data Extraction

Data extraction was performed by two researchers following the confirmation of the study inclusion. The extracted data included the first author’s information, publication year, sample size, age, height, weight, type of intervention, and post-intervention outcome measures (e.g., respiratory indicators, blood markers, and field test results). When necessary, the corresponding authors were contacted to resolve missing or additional data, ensuring accuracy.

### 2.5. Risk of Bias Assessment

The methodological quality of the included studies was independently assessed by two researchers using Review Manager 5.3 software. The risk of bias was evaluated across seven dimensions: randomized sequence generation (selection bias), allocation concealment (selection bias), the blinding of investigators and participants (performance bias), the blinding of study endpoints (measurement bias), the completeness of outcome data (attrition bias), selective reporting (reporting bias), and other biases. Each dimension was classified as “low risk,” “high risk,” or “unclear.” In cases of disagreement, a discussion among three reviewers was conducted to reach a consensus on the final assessment.

### 2.6. Data Analysis

Data analysis was performed using Review Manager 5.4 software to assess the effect of altitude training on athletes’ aerobic capacity. The effect size was expressed as the standardized mean difference (SMD) with a 95% confidence interval (CI). According to the Cochrane Handbook for Systematic Reviews of Interventions, either a fixed-effects model or a random-effects model was selected based on the actual effect of the intervention on outcome indicators. Heterogeneity was tested using the *p*-value and the I^2^ statistic, with I^2^ values of 25%, 50%, and 75% indicating low, medium, and high heterogeneity, respectively [29]. If high statistical heterogeneity was present (I^2^ ≥ 50%, *p* < 0.05), a random-effects model was applied; otherwise, a fixed-effects model was used. Meta-regression analyses were conducted to explore the sources of heterogeneity. Subgroup analyses, based on categorical variables such as the mode and duration of intervention, were performed to identify the most effective training types and durations for increasing hemoglobin levels. Sensitivity analyses were conducted to assess the robustness of the results by sequentially excluding each study and re-running the analysis. If excluding a study caused the estimate to exceed the 95% CI of the pooled effect, the study was considered to have significantly impacted the outcome, indicating potential bias. Finally, funnel plots were constructed, and Egger’s test was performed using Stata 17 to detect publication bias.

## 3. Results

### 3.1. Study Selection

A total of 11,305 articles were initially retrieved, and 86 were selected based on their title and abstract review. After applying the inclusion and exclusion criteria, 13 articles published between 2005 and 2024 were included in the analysis (Figure 1). Forty-four articles were excluded due to insufficient detail on intervention programs, sixteen were excluded for inadequate blinding, seven were excluded for being non-English or non-Chinese, and six were excluded for lacking the required outcome indicators. The final sample included 276 participants, with 153 in the experimental group and 123 in the control group. Participants were aged 18–35 years, with a mean age of approximately 21 years. Among the studies, five used the “Live-High-Train-Low” (LHTL) intervention [12,30,31,32,33], five used high altitude or simulated high altitude training [34,35,36,37,38], and three used the “Live-High-Train-High” (LHTH) intervention [39,40,41]. The majority of the interventions lasted between 3 to 4 weeks. Specifically, four studies had a 4-week intervention [30,31,32,40], eight studies had a 3-week intervention [12,33,34,35,37,38,39,41], and one study had an 8-week intervention [36].

### 3.2. Study Characteristics and Risk of Bias

**Figure 2:**
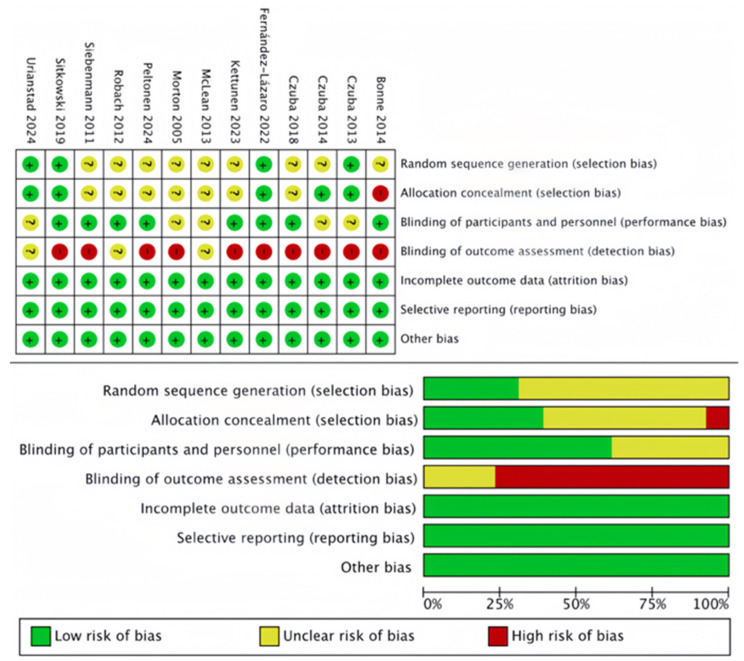
Risk of bias [12,30,31,32,33,34,35,36,37,38,39,40,41].

**Figure 3:**
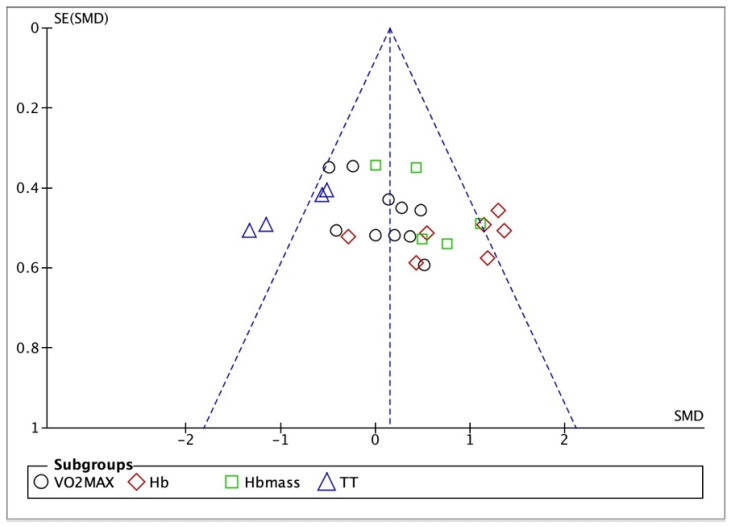
A funnel plot of VO_2_max, Hb, Hbmass, and time trial.

Of the 13 randomized controlled trials included, 153 participants were assigned to the experimental group, and 123 to the control group. Basic information for these 13 studies is provided in Table 1. The studies exhibited a relatively low risk of bias across various dimensions, indicating the high quality of the studies included in this meta-analysis (Figure 2). Furthermore, the funnel plot analysis did not reveal a significant publication bias, suggesting that the included studies were of a generally high quality, had substantial reference value, and met the requirements for secondary research (Figure 3).

### 3.3. Meta-Analysis Results

#### 3.3.1. Pooled Outcomes

**Figure 4:**
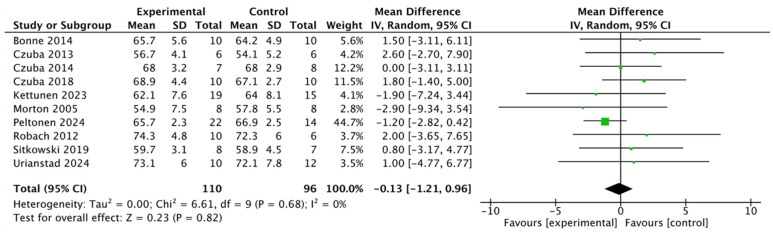
A forest plot of the effect of altitude training on athletes’ VO_2_Max [12,30,31,32,34,35,38,39,40,41].

Data from 10 studies, including a total of 206 participants, were analyzed to assess the effect of altitude training on VO_2_Max. The random-effects meta-analysis revealed a total effect size of −0.13 (95% CI: [−1.21, 0.96]) (Figure 4). There was no significant between-study heterogeneity (I^2^ = 0%, *p* = 0.68), and the results did not demonstrate a statistically significant difference.

**Figure 5:**
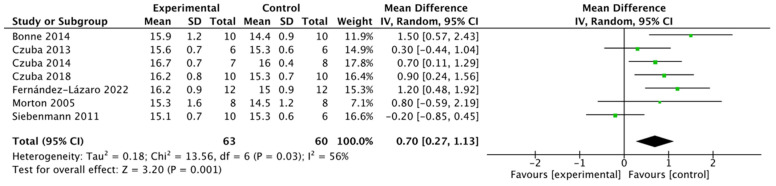
A forest plot of the effect of altitude training on athletes’ hemoglobin [30,33,34,35,36,38,39].

A second random-effects meta-analysis, evaluating hemoglobin levels, demonstrated that altitude training significantly increased hemoglobin levels. Data from seven studies, totaling 123 participants, yielded an effect size of 0.70 (95% CI: [0.27, 1.13]) (Figure 5). Moderate inter-study heterogeneity was found (I^2^ = 56%, *p* < 0.05), but the results were statistically significant.

**Figure 6:**
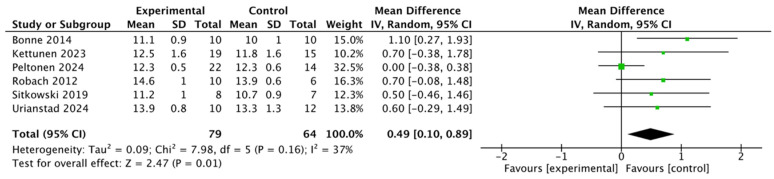
A forest plot of the effect of altitude training on athletes’ Hbmass [12,31,32,39,40,41].

For Hbmass, data from six studies, involving 143 participants, showed a total effect size of 0.49 (95% CI: [0.1, 0.89]) (Figure 6). Low inter-study heterogeneity (I^2^ = 37%, *p* > 0.05) was observed, and the results did not show a statistically significant difference.

**Figure 7:**
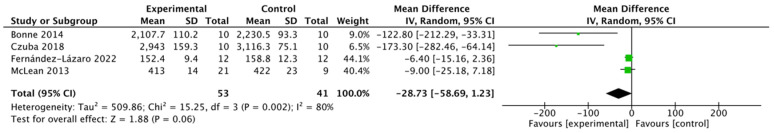
A forest plot of the effect of altitude training on athletes’ time trial [30,36,37,39].

Data from four studies, with 94 participants, assessed field test performance, showing an effect size of −28.73 (95% CI: [−58.69, 1.23]) (Figure 7). High inter-study heterogeneity was found (I^2^ = 80%, *p* < 0.05), but the results were statistically significant.

#### 3.3.2. Heterogeneity

Altitude training significantly affected athletes’ hemoglobin content, hemoglobin quality, and field test performance, while changes in maximal oxygen uptake (VO_2_Max) were not statistically significant. The variability in the results may be attributed to the absence of standardized protocols for assessing VO_2_max across the included studies. Different testing protocols, primarily including ramp tests [31,34,35] and step tests [12,30,32,33,38,39,40,41], were employed, which may account for discrepancies in the outcomes. Notably, one study on swimmers utilized an underwater step test. However, the existing literature has demonstrated that this technique provides valid VO_2_peak measurements, with a correlation coefficient of 0.92 when compared to treadmill or cycle ergometer tests [42]. In addition to the heterogeneity caused by different testing methods, variations in athlete performance levels may also impact the results. For example, one study on swimming selected 20 participants from the Danish Olympic Swimming Team and National Swimming Club [39], while three other studies set specific training experience requirements for their participants—5 [34], 6 [30], and 7 years [35], respectively. The remaining studies did not specify the training levels of their participants. As a result, differences in athlete performance levels could be a significant source of heterogeneity in the findings. These findings suggest that altitude training improves hemoglobin content and quality, which in turn enhances aerobic capacity and athletic performance, including field test outcomes.

##### Heterogeneity Test of Hemoglobin

The heterogeneity test for hemoglobin content revealed an I^2^ of 56%, indicating moderate heterogeneity. The source of this heterogeneity was further explored through sensitivity analyses, as the analysis was conducted using a random-effects model.

##### Heterogeneity Test of Hbmass

The heterogeneity test for hemoglobin mass revealed an I^2^ of 37%, indicating low heterogeneity. This suggests that altitude training effectively improves hemoglobin mass, and consequently enhances aerobic capacity in athletes.

##### Heterogeneity Test of Time Trial

The heterogeneity test for time trial revealed an SMD of −28.73 (95% CI: −58.69, 1.23, *p* = 0.002, I^2^ = 80%), indicating high heterogeneity (I^2^ > 50%). The source of this heterogeneity was explored through subsequent sensitivity analyses, as the analysis was conducted using a random-effects model. The heterogeneity observed in time trial tests may stem from variations in sports disciplines and testing methodologies. For instance, one study involving swimmers employed a 3000-m swim as the test [39], while another utilized a 30-km simulated cycling time trial [30]. Additionally, two other studies assessed 1000-m [36] and 2000-m [37] track running. These differences in sports disciplines impose distinct demands on athletes’ aerobic capacity, and the varying test distances may also affect the final results.

#### 3.3.3. Sensitivity Analysis

**Figure 8:**
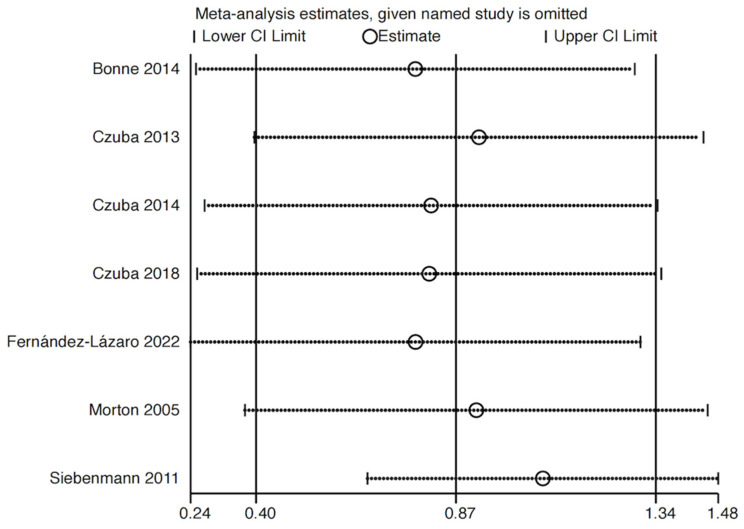
A sensitivity analysis of the effect of altitude on athletes’ hemoglobin [30,33,34,35,36,38,39].

**Figure 9:**
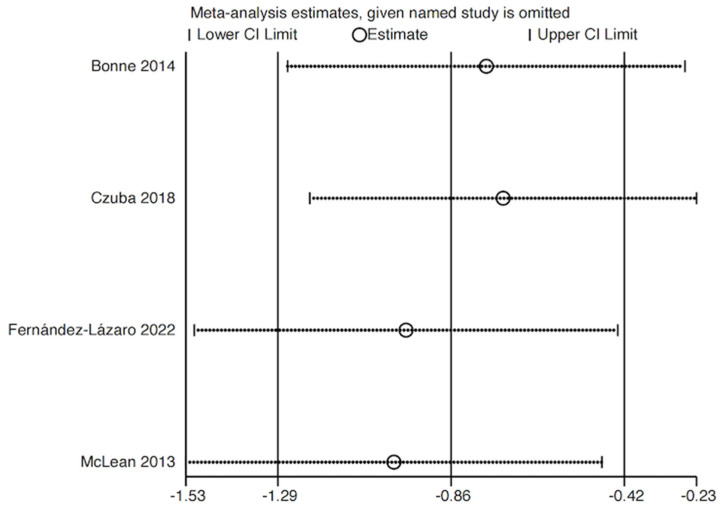
A sensitivity analysis of the effect of altitude on athletes’ time trial [30,36,37,39].

We conducted sensitivity analyses on the outcome measures of hemoglobin content and trial test performance, both of which exhibited a heterogeneity greater than 50% (I^2^ > 50%), to assess the potential impact of each study on the meta-analysis results. Using Stata 17 software, each study was re-analyzed by excluding them one at a time to determine the combined effect (Figure 8 and Figure 9). The sensitivity test results for hemoglobin content fluctuated around 0.87, while the results for trial test performance fluctuated around 0.86. These findings suggest that the results of our analysis are stable and have a high reference value.

#### 3.3.4. Bias Test

Given that the outcome indicators in this study were all continuous variables, Egger’s test was employed to assess the risk of bias (*p* > 0.05). The results indicated a low risk of bias, suggesting that our meta-analysis is reliable.

#### 3.3.5. Subgroup Analyses

**Figure 10:**
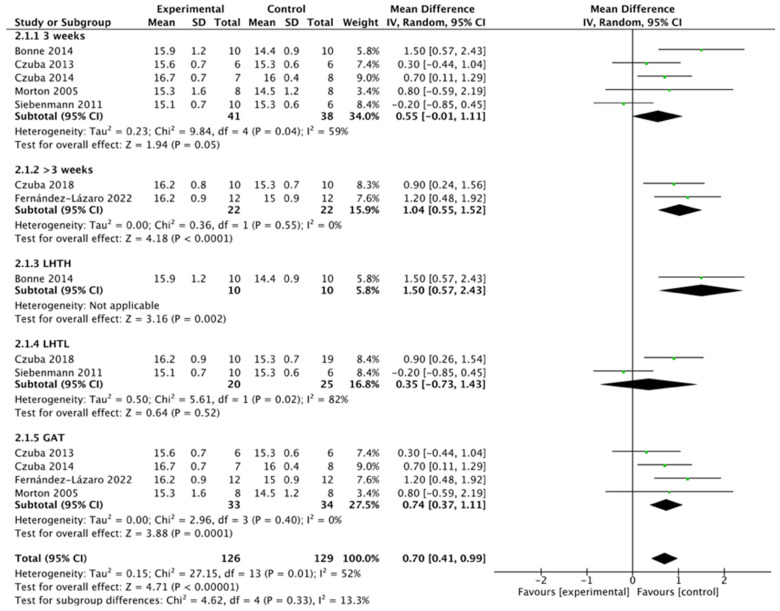
Subgroup analysis of the efforts of altitude training on the hemoglobin of athletes. Abbreviations: A: altitude training (intervention group) or after intervention; C: control group or before intervention; SD: standard deviation; LHTL: Live-High-Train-Low; LHTH: Live-High-Train-High; GAT: general altitude training [30,33,34,35,36,38,39].

We conducted a subgroup analysis to assess the effects of the altitude training mode and intervention duration on hemoglobin levels. The results indicated that the Live-High-Train-Low (LHTH) mode had a greater impact on hemoglobin levels compared to the Live-Low-Train-High (Lo-Hi) and General Altitude Training (GAT) modes, with the GAT mode showing a relatively smaller effect. Additionally, interventions lasting more than 3 weeks resulted in a greater effect on hemoglobin levels than those lasting 3 weeks. Since the literature included in this study showed no statistically significant effect of altitude training on VO_2_max, we did not perform a separate analysis for the effects of the training mode and intervention duration on VO_2_max levels (Figure 10).

## 4. Discussion

Thirteen studies were included in this systematic review and meta-analysis to examine the effects of altitude training on respiratory function, blood indicators, and trial test performance in athletes from various sports. The results of our meta-analysis revealed that altitude training significantly increased the hemoglobin content and quality in athletes, leading to improvements in aerobic capacity and, consequently, better trial test performance. However, our study found no significant impact of altitude training on maximal oxygen uptake (VO_2_max), and no heterogeneity was observed among the studies. In the subgroup analysis on hemoglobin content, we observed that interventions lasting more than three weeks had a greater effect on hemoglobin levels compared to those lasting three weeks. Additionally, the Live-High-Train-High (LHTH) training pattern appeared to have a more significant impact on hemoglobin content than the Live-High-Train-Low (LHTL) and General Altitude Training (GAT) modes, though further research is needed to confirm these findings.

### 4.1. Maximal Oxygen Uptake

The effects of the altitude training on athletes’ maximal oxygen uptake (VO_2_Max) differed from our initial hypothesis, which posited that altitude training would lead to greater improvements in VO_2_Max compared to sea level training. This also contrasts with conclusions drawn from previous systematic reviews [43]. Our meta-analysis showed no significant difference in the effect of altitude training on VO_2_Max in athletes compared to plains training (SMD = −0.13; 95% CI: −1.21, 0.96; I^2^ = 0%). Zelenovic’s research found similar results, showing no significant change in VO_2_MAX under both hypoxic and normoxic conditions [44]. VO_2_Max is strongly influenced by genetic factors, and late-stage training may not significantly enhance it [35,45]. While past studies have shown that increases in VO_2_Max are influenced by factors beyond genetics, such as the cardiorespiratory system’s ability to deliver oxygen to exercising muscles [46], improvements in blood oxygen content [19,47], and exercise economy [48], altitude training’s impact on VO_2_Max remains unclear. Additionally, high-intensity interval training (HIIT) has shown significant effects on hematological indices but a lesser impact on VO_2_Max [49]. The relatively small effect of altitude training on VO_2_Max in the athletes studied here may be attributed to the higher baseline training levels of the subjects, who had accumulated years of experience and exhibited a higher starting VO_2_Max compared to those in earlier studies [12,30,34,35] and the control group of plains training [31,36,40]. Athletes with a higher baseline VO_2_MAX tend to experience smaller improvements from training due to their limited capacity for further adaptation. Additionally, the principle of specificity suggests that the mode of training influences VO_2_MAX test results. Common VO_2_MAX testing equipment includes the treadmill, cycle ergometer, and rowing machine, and training in the same mode as the test generally leads to more effective adaptations, improving aerobic capacity and performance in that specific modality. Furthermore, the level of the subjects and the testing protocols can affect the outcomes. For instance, in Bonne’s 2014 study [39], 20 participants from the Danish Olympic Swim Team and the National Swimming Club were selected, but no differences in VO_2_MAX were observed between the groups, either before or after the intervention, possibly due to the elite athletes’ limited potential for further significant improvement. Additionally, variations in testing protocols, such as ramp tests [31,34,35] and step tests [12,30,32,33,38,39,40,41], employed in other studies may also explain discrepancies in results. Consequently, the increase in VO_2_Max in the experimental group was not statistically significant. Interestingly, the Live-High-Train-Low (LHTL) training model did not lead to a significant improvement in VO_2_Max [32]. However, one study reported an increase in other respiratory metrics, such as maximal minute ventilation, which rose from 165.1 ± 11.9 (L/min) before the intervention to 165.7 ± 12.7 (L/min) after the LHTL training [30].

### 4.2. Blood Indicator

The results of this meta-analysis align with findings from other studies that have shown an increase in both the quantity and quality of hemoglobin in athletes [12,13,14]. Altitude training may primarily affect an athlete’s aerobic capacity through a “hemato-logical mechanism”, where the hypoxic environment created by training increases the erythropoietin (EPO) concentration in the body, subsequently enhancing hemoglobin quantity and quality [50]. EPO release is triggered by three primary pathways: (1) a de-crease in hemoglobin levels, (2) hypoxia in the kidneys and lungs, and (3) direct adrenergic activation [51].

In situations involving hypoxia or an increased tissue oxygen demand, such as strenuous exercise, thin altitude air, or blood loss, the kidneys produce more EPO. This stimulates bone marrow hematopoiesis and accelerates the production and release of erythrocytes, alleviating the kidneys’ ischemic state—this is a negative feedback response by the body. Man’s study observed that altitude training led to an increase in EPO levels, which in turn significantly boosted hemoglobin concentration [52]. Six out of seven studies included in this meta-analysis showed that altitude training significantly increased the hemoglobin concentration per unit volume in athletes (*p* < 0.05, SMD = 0.7, 95% CI: 0.27, 1.13, Chi^2^ = 13.56, I^2^ = 56%). A sensitivity analysis using the one-by-one exclusion method revealed a stable result with fluctuations around 0.87.

In other experiments using the Live-High-Train-Low (LHTL) protocol, athletes exposed to hypoxia for more than 250 h (ranging from 300–500 h) demonstrated a significant increase in hemoglobin levels by 8–10% [53,54]. Besides the low oxygen environment, the daily training load of athletes is another crucial factor influencing erythrocyte counts during altitude training. Some studies have reported a significant elevation in the reticulocyte production index (RPI) from 30 min after an acute marathon to 6 days post-race (1.8% to 2.3%), which enhances the proliferative capacity of bone marrow to increase erythrocytes [55].

Altitude training not only affects hemoglobin quantity but also improves its quality. Five of the six studies examined showed an increase in hemoglobin quality (SMD = 0.49; 95% CI: 0.10, 0.89; I^2^ = 37%). Bonne’s study suggests that the stabilization of ferritin blood concentration (FBC) during altitude training is a key factor in improving hemoglobin quality, with 9 out of 10 swimmers experiencing a more than 3% increase in hemoglobin quality from baseline values [39]. Nummela further noted that individual differences in hemoglobin quality exist, and altitude training had a greater effect on hemoglobin quality in male athletes than in females [56]. Sitkowski’s study found that changes in hemoglobin mass were independent of initial ferritin or transferrin receptor concentrations, and that high-altitude hypoxic training could improve hematological parameters, even in athletes with relatively low baseline hemoglobin mass. High-altitude training stimulates erythropoiesis, which increases hemoglobin mass and enhances athletes’ aerobic capacity [12]. However, Robach’s study revealed no significant improvement in maximal oxygen uptake in athletes using the LHTL protocol [32]. Elite cyclists, for instance, did not experience significant gains in oxygen transport capacity from LHTL, likely due to their already high aerobic capacity, attributed to elevated hemoglobin mass and maximal oxygen uptake.

### 4.3. Trial Test Performance

The four studies included in this analysis used the 1000 m field run, 2000 m field run, 3000 m swim, and 30 km bike as the trial test events. A meta-analysis of these studies showed that altitude training was more effective in reducing athletes’ trial test times (*p* < 0.005, SMD = −28.73, 95% CI: −58.69, 1.23, Chi^2^ = 15.25, I^2^ = 80%). A sensitivity analysis using the one-by-one exclusion method revealed that the results fluctuated around −0.86, indicating that the findings were stable. The high heterogeneity (I^2^ = 80%) could be attributed to the varying test distances and events, as altitude training may affect different events and distances to varying degrees. Variations in test protocols and distances can significantly affect the physiological relevance of performance assessments by altering the load on the aerobic system. For instance, while swimming and cycling tests are strongly correlated with endurance performance, the intensity of aerobic stress depends on the test duration. Shorter tests (e.g., <10 min) often fail to fully engage the aerobic system, particularly in highly trained athletes, as they may not reach the intensity needed to activate maximal aerobic capacity. In contrast, longer tests (e.g., 20–30 min) impose greater demands on the aerobic system, offering a more accurate assessment of the effects of altitude training on field performance. For example, Fernández-Lázaro [36] employed a 1000 m time trial, with athletes completing it in under 3 min. This brief duration may not sufficiently activate the aerobic system, potentially diminishing the observable effects in the test results.

Similar findings were reported in a study by Wilhite et al., where subjects living at altitudes of 2000–2500 m above sea level and exposed to hypoxia for 20 h a day over 4 weeks showed a significant improvement in marathon performance in trial tests [57]. Dragos explored the effects of alternating hypoxic training on athletic performance and also found that the benefits of the LHTH training model were significantly greater than those of LLTL, which is consistent with the results of this study [58]. Previous studies have also indicated that altitude training enhances athletes’ quadriceps strength, contributing to an improvement of 3–10% in trial test performance. This enhancement may be related to the HIF-1-induced increase in the synthesis and secretion of insulin-like growth factor-1 (IGF-1), testosterone, and growth hormones [59]. Bonne’s study demonstrated that 3–4 weeks of altitude training at approximately 2200 m significantly improved 3000 m swimming performance [39].

Improved performance in the trial test reflects the athlete’s enhanced capacity, with the underlying factor being an increase in aerobic capacity, which includes an increase in blood volume and both the quantity and quality of hemoglobin.

### 4.4. Altitude and Duration

In research on the effects of high-altitude training, altitude and training duration are two critical variables that significantly influence the enhancement of athletes’ aerobic capacity. Existing studies demonstrate substantial variability in the impact of training at different altitudes on aerobic performance. It is widely recognized that an altitude range of 2000 to 2500 m represents an optimal training zone [60,61]. Within this range, athletes can effectively stimulate erythropoiesis and improve their maximal oxygen uptake while mitigating the excessive hypoxic stress that may impair training outcomes. In contrast, altitudes exceeding 3000 m, although potentially capable of further enhancing physiological adaptations, can impose heightened physiological strain and adversely affect recovery and performance.

Training duration is another essential factor that warrants careful consideration. Short-term high-altitude training (e.g., lasting a few days to one week) typically yields limited improvements in physical fitness. Conversely, long-term high-altitude training (e.g., extending over several weeks to months) has been shown to significantly enhance aerobic endurance. This disparity in outcomes may be attributable to the cumulative effects of physiological adaptation. A prolonged exposure to hypoxic conditions enables the body to gradually optimize its oxygen transport mechanisms, thereby enhancing the aerobic capacity upon return to low-altitude environments.

Notably, individual differences among athletes play a pivotal role in determining the effectiveness of altitude and training duration [62]. While some athletes exhibit a greater capacity for adaptation to high-altitude training, others may experience pronounced negative effects. Consequently, individualized adjustments to training altitude and duration are paramount to optimizing outcomes for each athlete.

Although this systematic review does not explicitly investigate the specific effects of altitude and training duration on athletes’ aerobic capacity, this area of inquiry merits further exploration. Future research should aim to identify the optimal combination of altitude and training duration to maximize improvements in aerobic performance, thereby contributing valuable insights to the field of sports science.

### 4.5. Limitation

The limitations identified underscore the complexity of the factors affecting the effectiveness of altitude training. These factors include athlete-specific variables, such as individual aerobic capacity, diet, rest, and mental health, as well as external elements like the characteristics of training environments and methodologies.

The need for future research is clear, particularly in addressing key operational questions, such as the optimal training mode, timing of return to sea level, and the applicability of altitude training across different sports.

## 5. Conclusions

This study evaluated the effects of altitude training on athletes’ maximal oxygen uptake, blood indicators, and trial test performance. The findings suggest that altitude training contributes to improvements in blood indicators, specifically hemoglobin quantity and quality, which in turn enhance aerobic work capacity. These improvements were reflected in better trial test performance.

However, the study found no significant enhancement in maximal oxygen uptake among higher-level athletes. While previous meta-analyses have validated the positive effects of altitude training on athletes’ performance, critical operational questions—such as the optimal training mode and the appropriate intervention duration—remain unanswered. This study provides more detailed recommendations. Specifically, it concludes that altitude training using a Live-High-Train-High (LHTH) mode and an intervention duration exceeding 3 weeks has a more pronounced effect on athletes’ blood indicators, leading to greater improvements in aerobic capacity and trial test performance.

## Figures and Tables

**Table 1:** Characteristics of all the studies included.

Authors	Sample Size (M/F,A/C)	Age (Years, Mean ± SD)	Height (m)	Body Mass (kg)	Type of Athletes	Intervention		Training Period	Performance Test
						A	C		
Bonne et al. (2014) [39]	20 (9/11,10/10)	20.9 ± 3.1	1.79 ± 0.06	72.8 ± 9.5	Swimming	LHTH	SLT	3 weeks	VO_2_max, Hb, TT
Czuba et al. (2013) [34]	12 (12/0,6/6)	22.0 ± 1.9	1.89 ± 0.61	83.9 ± 7.2	Basketball	GAT	SLT	3 weeks	Hb, VO_2_max
Czuba et al. (2014) [35]	15 (15/0,7/8)	25.0 ± 3.7	1.78 ± 0.05	67.8 ± 6.4	Biathlon	GAT	SLT	3 weeks	VO_2_max, Hb
Czuba et al. (2018) [30]	20 (—,10/10)	21.2 ± 3.5	1.79 ± 0.04	68.8 ± 4.3	Cyclist	LHTL	SLT	4 weeks	VO_2_max, Hb, TT
Fernández-Lázaro et al. (2022) [36]	24 (24/0,12/12)	25.7 ± 3.7	1.82 ± 0.49	74.0 ± 5.6	Athletes (middle- and long-distance)	GAT	SLT	8 weeks	TT, Hb
Kettunen et al. (2023) [31]	34 (14/20,19/15)	22.0 ± 4.0	—	69.1 ± 10.6	Cross-country skiers	LHTL	SLT	4 weeks	VO_2_max, Hb
McLean et al. (2013) [37]	30 (—,21/9)	23.0 ± 3.0	1.88 ± 0.08	88.0 ± 9.0	Football	GAT	SLT	3 weeks	TT, Hb
Morton et al. (2005) [38]	16 (16/0,8/8)	20.5 ± 0.8	1.78 ± 0.03	79.6 ± 10.2	Team sports players	GAT	SLT	3 weeks	VO_2_max, Hb
Peltonen et al. (2024) [40]	36 (21/15,22/14)	22.3 ± 1.0	1.79 ± 0.22	72.6 ± 2.4	Endurance athletes	LHTH	SLT	4 weeks	VO_2_max, Hb
Robach et al. (2012) [32]	16 (15/1,10/6)	29.0 ± 6.0	1.79 ± 0.08	69.0 ± 9.0	Cyclists, Triathlete	LHTL	SLT	4 weeks	VO_2_max, Hb
Siebenmann et al. (2011) [33]	16 (15/1,10/6)	29.0 ± 6.0	1.79 ± 0.08	69.0 ± 9.0	Cyclist	LHTL	SLT	3 weeks	VO_2_max, Hb
Sitkowski et al. (2019) [12]	15 (0/15,8/7)	20.5 ± 2.9	1.70 ± 0.05	60.2 ± 6.6	Track and field, Cyclists	LHTL	SLT	3 weeks	VO_2_max, Hb
Urianstad et al. (2024) [41]	22 (22/0,10/12)	26.0 ± 6.8	1.82 ± 0.49	74.0 ± 5.6	Cyclists	LHTH	SLT	3 weeks	VO_2_max, Hb

Abbreviations: M: male; F: female; A: altitude training (intervention group) or after intervention; C: control group or before intervention; SD: standard deviation; VO_2_max: maximal oxygen uptake; HB: hemoglobin; TT: time trial; LHTL: Live-High-Train-Low; LHTH: Live-High-Train-High; GAT: general altitude training; SLT: sea level training.

## Data Availability

The data from the study are included in the article, and inquiries can be directed to the corresponding authors.
